# Supplementary material for: The Genome of the American Dog Tick (Dermacentor variabilis)
Source: bioRxiv. 2025 Mar 14:2025.03.12.642860. Preprint. [Version 1] doi: 10.1101/2025.03.12.642860 (PMC11952394; doi:10.1101/2025.03.12.642860)
Supplement: 1 [file NIHPP2025.03.12.642860v1-supplement-1.pdf]

bioRxiv preprint doi: https://doi.org/10.1101/2025.03.12.642860; this version posted March 14, 2025. The copyright holder for this preprint (which was not certified by peer review) is the author/funder, who has granted bioRxiv a license to display the preprint in perpetuity. It is made available under aCC-BY 4.0 International license.

Supplementary Files

File S1: Supplementary methods

File S2: Transposon family database

File S3: Annotations in GFF format
